# An Examination to Explain the Mechanism of Employees’ Environment-Specific Behavior through CSR and Work Engagement from the Perspective of Stewardship Theory

**DOI:** 10.3390/ijerph18179370

**Published:** 2021-09-05

**Authors:** Shilong Wei, Muhammad Safdar Sial, Ubaldo Comite, Phung Anh Thu, Daniel Badulescu, József Popp

**Affiliations:** 1 School of Labor and Human Resources, Renmin University of China, Beijing 100872, China; shilongwei@ruc.edu.cn; 2 Department of Management Sciences, COMSATS University Islamabad (CUI), Islamabad 44000, Pakistan; safdarsial@comsats.edu.pk; 3 Department of Business Sciences, University Giustino Fortunato, 82100 Benevento, Italy; u.comite@unifortunato.eu; 4 Faculty of Finance and Accounting, Nguyen Tat Thanh University, 300A Nguyen Tat Thanh Street, District 4, Ho Chi Minh City 70000, Vietnam; 5 Department of Economics and Business, Faculty of Economic Sciences, University of Oradea, 410087 Oradea, Romania; dbadulescu@uoradea.ro; 6 Institute of Economic Sciences, Hungarian University of Agriculture and Life Sciences, 2100 Gödöllő, Hungary; popp.jozsef@uni-mate.hu

**Keywords:** CSR, SME sector, pro-environmental behavior, stewardship theory, work engagement

## Abstract

Corporate social responsibility (CSR) has emerged as an imperative for every sector globally. Yet, for a long time, the concept of CSR has been regarded from an organizational perspective. However, the importance of CSR in shaping the extra-role behavior of employees, such as their pro-environmental behavior (PEB-E), has been under-explored in the literature. Against this backdrop, the current study aims to investigate the impact of CSR on PEB-E with the mediating effect of work engagement (W.E) in the context of a emerging country. The study also employs stewardship theory as an alternate theory to explain the proposed relationships. The data of the current study were obtained from SME sector through a self-administered (paper-and-pencil method) questionnaire. A random sample of employees (*n* = 398) from different SMEs was selected and analyzed through structural equation modeling. The results of the current survey revealed that CSR directly and indirectly, via W.E, predicts PEB-E positively. The findings of the current study will be helpful for policymakers to understand that well-planned CSR activities, not only create positive repute for an SME, but also provide the underlying justification to its employees to be engaged in different environment-specific behaviors.

## 1. Introduction

### 1.1. Background and Rationale

Concerns about the environment and environment-related problems are growing among the public more than ever before. Global environmental issues pertinent to climate change, i.e., ozone depletion, water scarcity, deprived ecosystems, and forest depletion are some of the factors that have contributed to the growing concern among stakeholders. The role of businesses in the field of environmental protection becomes clearer, SMEs need to be more accountable in ensuring a sustainable environment [1]. Businesses need to streamline their business activities by considering environmental protection and the proper management of natural resources. In today’s dynamic business world, organizations are acknowledging the importance of their employees and supporting them to be engaged in different activities and contribute to the overall performance of the organization [2]. Likewise, there is a general consensus among scholars that employees of an organization are key factors for enhancing the environmental performance of an organization [3,4,5]. Therefore, organizations are exploring different ways to shape the behavior of employees, especially the pro-environmental behavior of employees (PEB-E). 

In this regard, scholars have recently noted the importance of corporate social responsibility (CSR) to shape the behavior of employees towards the environment [6]. This is the basis for the noticeable surge in CSR studies influencing PEB-E [7,8,9]. The concept of CSR has a long history, but its boundaries are still evolving even in 2021 [10]. CSR is beneficial for all business industries. Businesses are increasingly recognizing the importance of CSR as a way to meet important social challenges, in order to achieve the objective of sustainable development [11]. These social challenges need to be addressed effectively, and a shared effort from all stakeholders is required. Practicing CSR is difficult, and faces significant problems in the context of business environments that are characterized by rapid technological changes, globalization, and stiff competitive situation that prevails almost in every sector [12]. Due to such complexities, organizations are required to adopt a learning orientation to remain competitive in their relevant business sector.

In the beginning, the concept of CSR was limited to the extent of philanthropic orientation [13,14]. However, at later stages, the organizations learned that CSR may be tapped to achieve different organizational related outcomes, including organizational performance [15,16,17] and others [18,19]. In this context, the learning repository of the organizations continued to help businesses to broaden the spectrum of CSR to new horizons. This is why, presently, CSR is regarded as a multitude of phenomena that help businesses achieve different organizational objectives. In this regard, some recent scholars have argued that CSR activities of an organization can assist in motivating employees to engage in different extra roles to induce organizational performance. For example, Raza et al. [20] show that a positive relationship exists between CSR and PEB-E. 

In existing literature, different scholars have focused on the organizational outcomes of PEB-E [21,22], while neglecting the underlying mechanism that urges employees to be engaged in environment-related behaviors. Here, we take a different stance by arguing that, although the importance of PEB-E in achieving various organizational objectives (and especially environmental objectives) cannot be denied, understanding the mechanism for engagement of employees in such behaviors is more important. Therefore, the current analysis attempts to explore the underlying mechanism for the involvement of employees in pro-environmental behavior through employees’ CSR perception and with the mediating effect of work engagement (WE). 

**Figure 1:**
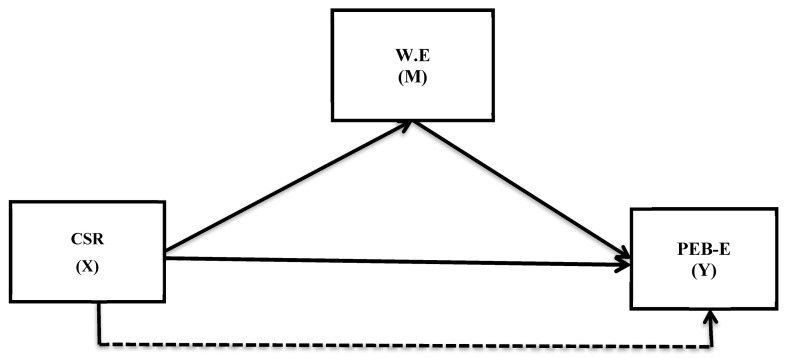
The proposed research model: Corporate social responsibility (CSR) = the independent variable (X), work engagement (W.E) = the mediating variable (M), and pro-environmental behavior of employees (PEB-E) = the dependent variable (Y).

The proposed model (Figure 1) of the current analysis was employed in the small and medium enterprise (SME) sector. We considered this sector to be suitable for the current analysis due to the following reasons. First, the vulnerable climatic conditions in previous years have placed the country in the list of top ten countries most vulnerable to changing climatic conditions [23]. In this context, industrial pollution is one of the major reasons that significantly contribute to environmental damage in some emerging countries [24]. Surprisingly, scholars have largely addressed this issue of climate vulnerability in the context of large enterprises, while the SME sector was neglected in this respect [25]. We argue, here, that the SME sector as a whole produces a significant amount of pollution that should not be ignored. The reason for our argument lies in the logic that the SME sector is the major industrial contributor in the country; this sector contributes up to 60% of the country’s GDP [26]. Therefore, the relevance of this sector in environmental dilapidation cannot be denied. The neglect of this sector by contemporary scholars is something that motivates us to carry out this research in the SME sector of a country that is vulnerable to climatic conditions. Second, the SME sector is a highly labor-intensive sector that provides nearly 78% of employment to the non-agricultural labor force. Therefore, non-agricultural labor sector employs millions of people, and if employee attitudes in this sector improve towards greater environmental protection, the country can hopefully achieve a better and sustainable future. 

The current analysis offers some important literary contributions in the available literature of organizational behavior and CSR. For example, the current analysis highlights the importance of CSR in shaping the behavior of employees for a better and more sustainable future. In this regard, the majority of the previous studies in the domain of CSR were conducted from other perspectives rather than considering the employees to shape their pro-environmental behavior through their CSR perceptions [27,28,29,30]. Furthermore, the current analysis also significantly contributes to the available literature as it emphasizes the underlying mechanism of CSR and PEB-E relationship. Whereas previous studies have largely explored the outcomes of PEB-E on organizations. Furthermore, we introduce W.E as mediator to explain the relationship between CSR and PEB-E. The vital role of W.E as a mediator to explain employees’ involvement in different extra roles is pre-established [31,32]. In this context, we mention that PEB-E is also an extra-role behavior, but the mediating effect of W.E in explaining PEB-E is under-explored in the existing literature. 

### 1.2. Theoretical Underpinning

The current analysis employs stewardship theory as a grounding to explain the proposed relationships of variables and develop different hypotheses. This theory states that individuals are motivated to work for others or organizations to fulfill the duties and responsibilities for which they are entrusted. The stewardship theory was proposed by Donaldson and Davis [33], who posited that individuals are collective-minded and pro-organizational instead of being individualistic, and thus, they are expected to work for the collective interest of a group or society. This theory has been used by different scholars to understand the motivations behind the behavior of individuals [6,34,35]. Following this theory, we argue that individuals in the workplace are stewards who assume the responsibility of taking care of resources. Therefore, workers as stewards perform their duty in a careful manner, even for those tasks for which they are not officially responsible. As a steward, a worker suppresses his or her personal interests and keeps the collective interest of the group (organization and society) at the forefront. Recently, different scholars have linked this theory with corporate governance by contending that individuals in the workplace are stewards who are expected to act in ways for the benefits of others [36,37]. 

The notion of stewardship joins the CSR lexicon considerably, as this concept is well-linked with CSR through “shared responsibility” for all stakeholders, in order to perform their job in ways that produce larger benefits for the society [38]. More specifically, the concept of environmental stewardship is at the heart of CSR philosophy. Under the concept of environmental stewardship, organizations are responsible of taking care of the interests of future generations, as each generation is expected to pass on to the future generations, for which they were entrusted, in an ethical way [39]. Therefore, stewardship theory helps our understanding of the underlying mechanism of psychological factors that shape specific behavior of individuals especially their discretionary behaviors [40]. In this regard, we argue that the CSR perception of employees about their organization is a motivating factor that inculcates in them the notion of stewardship and they are expected to practice this stewardship orientation on their own. 

## 2. Development of Hypotheses

### 2.1. CSR and Pro-Environmental Behavior of Employees

Employees are also one of the key actors in an organization to support and promote, workplace policies [41]. As a result, they influence and are influenced by CSR policies and procedures at all levels of engagement. Theoretically, it is established that a positive relationship exists between CSR orientation of an organization and employees’ behavior [42,43]. CSR activities, which are the social and environmental obligations of an organization for the society, can be helpful in shaping the pertinent behavior of employees [44]. CSR activities may also show greater interest for employees to fulfill their needs [45]. Moreover, researchers have argued that CSR actions can promote the behavioral intentions of employees in a favorable manner for an organization [46]. 

Organizations around the world are paying close attention to preserve the natural environment. This is why companies are moving forward and searching for new ways of sustainable development. In this regard, some of the effective solutions used by businesses involve engaging and motivating employees in PEB, which are considered the behaviors of an individual in reducing one’s negative impact on the environment [47]. PEB-E is a kind of discretionary behavior that is not an official requirement of an organization from an employee. However, the engagement of employees in such behavior is very important for the organization and society. Instances of PEB-E in the workplace include ideas for improving the environment, recycling waste, saving energy by turning off unneeded lights, printing paper on both sides, reducing the use of waste, and using stairs instead of elevators [7]. Many researchers have identified the potential of CSR in shaping employee discretionary behavior towards the organization [48,49,50]. Organizations incorporating CSR strategies have various intangible resources, including a humanistic culture, which makes an organization desirable for employees to have a sense of obligation to reciprocate through positive voluntary actions, such as the involvement in different PEB.

CSR activities build social resilience because CSR provides multiple benefits to all stakeholders including the employees. Therefore, when employees receive these benefits in the form of work-life balance, green education, and training, they are expected to return such benefits by displaying different extra-role behavior for the betterment of the organization and society, and one such role is PEB-E [51]. Employees receive information about their organization’s CSR activities through different sources, including emails, newsletters, and seminars. Following the stewardship theory, an organization’s CSR engagement is taken well by employees, and it inculcates that, like their organization, they are also stewards in preserving nature for future generations. Therefore, they become more ethically responsible and are expected to be more engaged in PEB. This argument receives considerable support from various recent studies [52,53,54]. On this basis, the following hypothesis is framed.

**Hypothesis** **1** **(H1):** *There exists a positive relationship between CSR and the pro-environmental behavior of employees.*

### 2.2. CSR and Work Engagement 

W.E has gained a lot of attention from scholars in recent days, as it has been considered an important factor in the success of the workplace [55]. Cesário and Chambel [56] acknowledged that W.E is one of the most important components of any successful organization. A workplace that is characterized by disengaged workers is unlikely to produce effective outcomes for an organization. In fact, a recent survey revealed that out of five billion people globally, only 1.4 billion have a reasonable job and among them, 16% reported that they are engaged workers [57]. Several scholars have noted the importance of W.E for better and sustainable development [58,59]. There are different factors that influence the W.E of employees at the workplace, and organizational systems, culture and structures are some of the most important factors for engaging workers in an organization [60]. Some recent researchers have posited that inappropriate and unscrupulous corporate behavior leads to frustration, discouragement, and layoffs of employees [61]. Conversely, employees serving in socially responsible organizations receive rewards in the form of intrinsic motivation, self-confidence, including W.E and satisfaction [62,63]. Chaudhary and Akhouri [64] posit that W.E is a precursor for employees to be engaged in different extra-role behaviors. Along with the passage of time, W.E has emerged as a very important phenomenon for organizations, given that, by involving employees, organizations are better able to solve different challenges encountered by businesses in recent times [65]. Engaged employees are expected to be fully absorbed in their work and engage themselves in behaviors that promote overall organizational efficiency. In this respect, a socially responsible organization is likely to create an organizational culture in which workers feel comfortable, flexible, and motivated, not only to perform their formal work obligations in an efficient manner, but they are also expected to go beyond their formal limits to support their socially-responsible organization [20]. 

CSR is a priority agenda for all organizations in the current age, regardless of the size and capacity of the activities an enterprise intends to contribute to the development of the community and society as a whole. Importantly, contemporary enterprises want to be seen as contributing to the compilation of the best practices in their business operations [66]. From contributing to hygiene planning to educating disadvantaged children, organizations in the present age leave no stone behind in rebuilding society. More importantly, according to a study, CSR is the third most important factor in keeping employees engaged in the workplace [67]. The report conducted by Kenexa Research Institute further confirmed the significance of CSR by asserting that employees working in a socially responsible organization feel pride and are engaged to work for such an employer. Further, such employees also recommend to others that the organization is a desirable place to work [68].

A workplace characterized by the philosophy of CSR inculcates in its employees the sense of caring for others, and employees become convinced that if their organization takes care of the interest of all stakeholders, they should also support their organization in this noble cause. Therefore, in a socially responsible organization, workers are fully engaged in their work and bring their whole selves to the workplace [69]. In like manner, Glavas and Piderit [70] proposed that CSR and W.E are positively related in a sense that an employee’s perception regarding CSR engagement of his or her organization inculcates a greater sense of meaning and a congruence between employee values and organizational values. Similarly, the same finding was reported by Caligiuri, et al. [71], as they postulated that W.E is an outcome of employees’ CSR perception about their organization. More specifically, the CSR orientation of an organization motivates itself to go beyond the formal obligations and pay an added attention to the betterment of society, the environment, and the employees [63]. Therefore, in response, employees become motivated as being members of an organization that is socially responsible and try to engage themselves more fully while are performing different tasks in an organization. Therefore, the following hypothesis is proposed:

**Hypothesis** **2** **(H2):** *There exists a positive relationship between CSR and work engagement.*

### 2.3. Work Engagement and Pro-Environmental Behavior of Employees

Some scholars have argued that the environmental concern of an enterprise, including a strategic vision is a critical determinant of employee involvement in environmental activities [72,73]. W.E has received increasing interest from researchers recently, and remains a very important and timely issue as it is considered to be the most important factor in occupational well-being [74]. W.E is important for organizations, given that through engaged employees, organizations are able to cope with the current challenges [65]. W.E is one of the most important factors in the success of any organization. Kahn [75], described W.E as a cognitive, emotional, and physical condition of an employee. The cognitive factor encompasses employee beliefs about organizational leadership and the context (workplace environment) in which it operates. The emotional state affects employees’ attitudes towards the organization, such as attachment and feelings of pride with an organization. The physical aspect is the way in which workers use their potential to achieve tasks and work-related goals while performing different tasks in an organization. 

Researchers in the field of organizational management contended that misconduct and unethical conduct of a firm lead to employee dissatisfaction, hopelessness, and frustration [61]. Whereas, employees of responsible enterprises receive awards in the form of internal motivation, confidence and performance, participation, and satisfaction [76]. When firms participate in role-based identification, such as public service, community engagement, adopting green approaches and policies, and engaging different stakeholders in their business activities, these firms are regarded as role-models by the employees. To put it simply, this notion of a role-model from socially responsible organizations is likely to have an impact on the behavior of employees, and they are encouraged to redefine their behavior to support their organization.

Once the workers have identified them with the organization as an outcome of its CSR commitment, they then put energy, dedication, and are fully engaged in their work [77]. Furthermore, by responding to community-services, a firm sends a clear message to all stakeholders including the workforce, about its commitment to serving the environment and the community beyond a bottom line (financial) objective. Therefore, employees are more likely to respond to CSR actions because of their value congruence with the organization. An employee’s understanding of a firm’s CSR activities will lead him or her to be a better and more engaged worker [78]. Likewise, agreeing with Brewer [79] and Steffens et al. [80], we argue that socially responsible organizations value the diversity of employees by their involvement in environment-specific behaviors and also encourage them to help others contribute to the environmental goals of the organization.

Employees can improve the environmental footprint of their organization by engaging in their work [51]. The engaged employees demonstrate resilience to the environment through eco-friendly initiatives, as well as helping other employees and peers to understand the green/ethical concerns associated with the sustainable goals of the organization and society [81]. Employee engagement in the workplace reflects their positive attitude and purpose leading to various unscripted guidelines towards the environment. Therefore, organizations that encourage workers to negotiate their work in relation to the environment. Therefore, the following hypothesis is proposed.

**Hypothesis** **3** **(H3):** *There exists a positive relationship between work engagement and the pro-environmental behavior of employees.*

### 2.4. CSR, Work Engagement and Pro-Environmental Behavior of Employees

CSR perception of employees about their organization has been reported by several scholars to induce environment-specific behaviors [7,50]. When employees observe that their organization is paying a serious concern towards mitigating its environmental footprint, they are also convinced to stand by their organization in this social cause. A stream of researchers has linked CSR with the pro-environmental behavior of the employees [8,51,52]. Moreover, the CSR perception of employees influences their behavioral intention of taking care of society and the environment. Put simply, in line with the stewardship theory, the CSR orientation of an organization inculcates a sense of stewardship among the employees and each employee is motivated to consider himself or herself as a steward in preserving nature for future generations. This entire process translates to better employee behavior towards the environment. Moreover, we like to refer to the study of Popescu [82], who argued that the beliefs, values, and attitudes of an individual significantly influence his or her behavioral intentions. Specifically, social context (workplace) is also critical in shaping individual behavior. Given that the workplace environment has a significant potential in influencing the beliefs, attitudes, and value systems of an individual, we expect that a socially responsible organization is one that provides a social context to its workforce in which they can adopt eco-friendly behaviors. More specifically, the CSR orientation of an organization has the potential to enhance the motivations of employees and their intentions for organizational support, and to alter their existing habitual behavior into pro-environmental behavior. 

To conclude, the CSR perception of an organization is expected to produce an engaged workforce for the organization. Given that the engaged workers bring their whole selves to the workplace and are fully absorbed in their job, they are expected to go beyond their formal working obligations to support their organizations. Several other scholars have also reported W.E as the mediating variable for explaining the extra-role behavior of the employees [31,83]. The pro-environmental behaviors of employees are also extra-role behaviors, so the engaged workers are expected to have a positive intention to preserve nature and the environment. More specifically, the value congruence of engaged employees with their socially responsible organization also urges them to take care of all stakeholders. Therefore, we propose the following hypotheses.

**Hypothesis** **4** **(H4):** *Work engagement mediates the relationship between CSR and pro-environmental behaviors of employees.*

## 3. Method

### 3.1. Sample and Population

The SME sector constitutes more than 90% of the total number of businesses and the contribution of this sector to the country’s Grand Domestic Product (GDP) is more than 60% [84]. Despite the fact that the SME sector is a significant economic contributor to the country, it is also important to note that this sector is also attributed to the widespread industrial pollution in the country. Equally important to point here is that the SME sector constitutes more than twenty million enterprises [85]. Therefore, this sector employs huge number of individuals, and their responsibility toward the environment is a matter of serious concern if the country wants to achieve a better and sustainable future. 

Cities constitute a multi-million population; however, this population is facing different climatic issues, including poor air quality index, contaminated water, and others. In fact, the city from some emerging countries has been reported as one of the most polluted cities in the world [86]. Second, current city also constitutes a significant proportion of SMEs as the industrial identification, better accessibility, ease of doing business, and the availability of a skilled labor force, are some factors that make a favorable place for SMEs. 

### 3.2. Procedure

In order to collect the data from the SME sector, we first identified the SMEs which were involved in some sort of CSR activities. This was done by visiting different SMEs and asking the management for their involvement in CSR activities. After this identification, we prepared an initial list of enterprises to be contacted with the request to facilitate us in the process of data collection. Next, we contacted the listed enterprises to indicate their willingness for their employees to participate in the questionnaires. The enterprises that showed their willingness were approached by the authors for finalizing the dates and timing to collect the data from their employees. It should be noted that the outbreak of COVID-19 posed some serious challenges for us during data collection. For example, we had to follow strict entry protocols for safe entry in the selected SMEs. Similarly, we had to stay extra hours in some SMEs due to restricted socialization. The data were collected between January 2021 and February 2021.

The data for the current analysis were collected from 29 SMEs from different sectors (Table 1). In this respect, we distributed 600 plus surveys among different employees of these SMEs, including managers and non-managers (employees with non-managerial positions). Prior to requesting an employee fill out the questionnaire, we followed the ethical guidelines of the Helsinki Declaration, in order to fulfill the ethical requirement for the current analysis. The participants were given a choice to quit the survey at any stage if he or she was uncomfortable in providing the required data. Moreover, we also received informed consent from each respondent to participate in the survey. For this reason, we attached a separate page along with the questionnaire for the convenience of the respondents. Among 600 plus questionnaires, we received a total of 398 questionnaires, which were included in the final dataset of analysis. Therefore, this survey produced a response rate close to 67%. The demographic profile of the sample is shown in Table 1. 

### 3.3. Instrument

We used already-established scales to operationalize the constructs of the current analysis. Therefore, the reliability and validity of the scales were already established. In this regard, we adapted the CSR scale from the study of Turker [87]. This scale has been widely used by various scholars to record CSR perceptions of employees, for example, Raza, et al. [20] used this scale to measure employees’ CSR perception in the context of the hotel sector. Whereas, in their recent study, Tian and Robertson [51] used this scale to measure CSR perception of employees in the large and medium casino industry of China. This scale was comprised of 12-items among which a sample item was “This enterprise targets sustainable growth, which considers future generations”. The scale of work engagement was adapted from the study of Schaufeli, et al. [88]. This scale is used by several extant researchers to measure the construct of W.E. For instance, Chaudhary and Akhouri [31] employed this scale for measuring W.E in the context of IT firms of India. Moreover, Nazir and Islam [61] used the same scale of W.E in the hospitality sector of India. The scale of W.E was comprised of 9-items. A sample item was “I am proud of the work that I do”. Lastly, the scale of pro-environmental behavior was employed from the study of Robertson and Barling [89], which is a well-known scale to measure the construct of pro-environmental behavior. Previous researchers like Latif and Aziz [90] used this scale in the healthcare sector. While, Afsar et al. [91] used this scale in various industrial sectors. This scale was comprised of 7-items among which a sample item was “I turn lights off when not in use”. We used a five-point Likert scale to record the ratings of respondents. 

### 3.4. Statistical Tests

The current study employed different statistical tests for validating the dataset and for testing the hypotheses. For example, confirmatory factor analysis (CFA) was used for construct evaluation, descriptive statistics was used to describe the data, and correlation analysis was used to assess the level of correlation between different constructs. Moreover, the study also employed structural equation modeling (SEM) to test the hypotheses of the current survey. Table 1 provides the demographic detail of the sample. 

## 4. Results 

### 4.1. Common Method Bias

Table A1 shown in Appendix A. Common method bias (CMB) is a matter of concern in surveys where all the information of variables is collected from the same respondent [92]. Due to the existence of CMB, it is assumed that the variation in responses is presumed because of the instrument rather than the predisposition of the sample that is more important for every research project. As the current investigation collected data for all independent and dependent variables at a specific point in time, thus there may be the existence of CMB. Therefore, we employed a single factor analysis, as proposed by Harman [93]. The results of a single factor analysis indicate the presence of a single factor that explains a large variation (50% or above), then the whole output of a research project becomes unreliable as the data suffer from this issue of CMB. Fortunately, in the current analysis, the output of single factor analysis produced acceptable results, as the most dominant factor explained less than 43% of the total variation. Based on this evidence, we established that the potential issue of CMB in the current case does not require any correction. The output of single factor analysis is given in Table 2.

### 4.2. Factor Analysis, Validity, and the Reliability

After validating that CMB is not a potential concern in the current scenario, we moved forward to the next phase in data analysis. On this point, we performed several tests, for example, exploratory factor analysis, convergent validity, and reliability tests. The results of the analysis have been reported in Table 3. As evident from Table 2, the results of exploratory factor analysis (principal component analysis) revealed that all the item loadings produced sufficient results, as the factor loadings were beyond the cut-off level (λ > 0.5). In this regard, only one item of PEB-E produced weak loading (PEB-E3 with λ = 0.38), thus we dropped this item from further analysis. The exploratory factor analysis was carried out by using the varimax rotation method. We also tested the convergent validity of our instrument by calculating average variance extracted (AVE) by using the following formula:

(1) $$\mathrm{AVE}=\frac{\sum_{\dot{i}=i}^{k}\lambda_{i}^{2}}{\sum_{\dot{i}=i}^{k}\lambda_{i}^{2}+\sum_{i=1}^{k}var\left(\varepsilon i\right)}$$

The AVE value for a construct indicates what percentage of variation is caused by the construct in relation to the measurement error term [94]. An AVE value greater than 0.5, is desirable as it is an indicator that more than 50% of the variance is due to the construct. In this context, the AVE values for all constructs were more than 0.5 and we were in line with Gefen et al. [95] who mentioned that the convergent validity for a construct is maintained if that construct produces an AVE that is greater than 0.5.

We also calculated the composite reliability (CR) for each construct. In order to do so, we used the following formula to calculate CR for each of our constructs. The general rule is that the CR value for a construct should be 0.7 or above. In this regard, one can see from Table 3 that all the constructs were producing CR values more than this threshold level of 0.7:

(2) $$\mathrm{CR}=\frac{\left(\sum \lambda i\right)2}{(\sum \lambda i)2+\sum var\left(\varepsilon i\right)}$$

Next, we employed correlation analysis, discriminant validity test, and model fit indices (during confirmatory factor analysis). These results are presented in Table 4. According to these results, it is evident that our three constructs are positively correlated. As a case, it can be seen that the value of correlation between PEB-E and W.E is 0.33, which is positive and implies that these constructs are positively correlated. Furthermore, these values of correlations are within the moderate ranges, which means that there is an absence of the potential issue of multicollinearity. Moreover, the values of maximum shared variance (MSV) and average shared variances are smaller than the corresponding values of AVE. For instance, AVE for CSR is 0.76, which is far beyond the values of MSV and ASV (0.17, 0.16). Moreover, we also tested for Heterotrait Monotrait Method (HTMT) to further validate discriminant validity. The results (Table 4) of HTMT also indicated that discriminant validity is established in the current case [96,97]. Lastly, the measurement model results produced significant results (*χ*^2^ = 3073.932, *df* = 788, *p* < 0.01 *χ*^2^/*df* = 3.886, RMSEA = 0.058, CFI = 0.932, NFI = 0.912). In this regard, *χ*^2^/*df* is smaller than the cut-off range of 0.5 which implies that there is a good fit between data and the theory [98]. Similarly, the values of CFI and NFI were above 0.9, validating that both values are acceptable. Finally, the RMSEA value was also significant (<0.06) for the measurement model. 

### 4.3. Hypotheses Testing

We validated the hypotheses of the current survey by employing the structural equation modeling (SEM) technique in AMOS software. In this regard, we were in agreement with other authors [99,100,101], who acknowledged the superiority of SEM in dealing the complex models. SEM has become a modern data analysis technique as it offers more sophisticated and superior data analysis techniques in comparison to the conventional regression in SPSS software. The hypotheses were validated by employing a two-step process in SEM. In this context, the first step was employed to measure the direct effects of predictors on the criterion. In this step, W.E was not acknowledged as a mediating construct because we were interested to check the direct effects. We have reported the output of the direct effect model in Table 5. According to the results, first three hypotheses (H1, H2, and H3) of the current analysis were significant (*β*1 = 0.36, *β*2 = 0.32, *β*3 = 0.26, *p* < 0.05). We established these findings based on the beta values, which were positive for all three cases and the *p*-values (<0.05) along with the values of upper limit confidence interval (ULCI) and lower limit confidence (LLCI) interval, as both of these values did not include a zero. Finally, the model fit indices were also significant (*χ*^2^ = 2738.622, *df* = 843, *p* < 0.01 *χ*^2^/*df* = 3.25, RMSEA = 0.055, CFI = 0.937, NFI = 0.914). 

In the second step, we introduced W.E as a mediating construct in the structural model and observed the output to verify that either W.E is mediating between CSR and PEB-E. To achieve this, we employed the bootstrapping method by choosing a large bootstrapping sample of 2000. The results have been reported in Table 6 for the readers. The bootstrapping results revealed that there is a partial mediation effect of W.E between CSR and PEB-E. We established these results because the value of beta was reduced (*β*1 = 0.36, *β*4 = 0.083), but remained significant which implies that there is a partial mediation effect. Moreover, the mediating effect was explaining more than 23% of the total variation in PEB-E which is positive and significant. Lastly, the results of model fit indices were most appropriate for our mediated model which implies that the data fitting the model in more appropriate manner which is positive point to indicate that our proposed model is appropriate (*χ*^2^ = 2311.492, *df* = 872, *p* < 0.01 *χ*^2^/*df* = 2.65, RMSEA = 0.048, CFI = 0.947, NFI = 0.919).

## 5. Discussion

The current analysis was carried out to serve two specific purposes. First, to explore the underlying mechanism of employees’ engagement in pro-environmental behavior at the workplace through CSR and work engagement. Second, the current study also attempted to link stewardship theory as an underlying theory to explain the psychological mechanism of individuals in taking caring of the things for which they are entrusted. The findings of the current survey revealed that employees of an organization feel alleviated when they are serving a socially responsible organization. In fact, when employees observe that their organization is showing concern for the betterment of society and the environment, they then believe that their organization is a caring organization, which intends not only to take care of the economic benefits but also intends to pursue its societal responsibility. Therefore, employees also develop the same feelings on their part too, and they want to support their organization to be more engaged in different societal activities. Thus, employees engage themselves in responsible behaviors by showing a greater sense of responsibility. One such responsible behavior is their engagement in pro-environmental behavior. CSR perception of employees about their organization leads them to practice pro-environmental behavior. The current study is not the first to propose this relationship as some recent scholars have also concluded that employees’ positive CSR perception and their pro-environmental behavior are positively linked [50,53]. Moreover, the current study also validates that work engagement is a phenomenon that serves the relationship of CSR and PEB-E as a potential mediator. The underlying explanation for this association is that engaged employees bring their whole selves to the workplace and they want to serve their organization even beyond the formal limits. Engaged employees are fully absorbed in their work and they feel themselves a part of their organization, and thus they put sincere efforts towards the success of their organization. Studies have consistently reported that engaged employees are better expected to participate in extra-role behavior [31,102,103], unlike non-engaged employees who join an organization just to fulfill their monetary and professional purposes. Moreover, the mediating effect of work engagement to shape the employees’ behavior also receives support from the extant literature [104,105]. 

Therefore, the study confirms that CSR perception of employees for their organization predicts employees’ pro-environmental behaviors with work engagement as a mediator. The underlying mechanism of employees’ participation in different environment-specific behavior can also be explained from the perspective of stewardship theory. This theory argues that individuals are stewards who are assumed to take care of others and pass on carefully to the future generation the assets for which they were entrusted. In the context of the current study’s objectives, the concept of natural stewardship is well fitted here to explain why individuals at the workplace are expected to be engaged in pro-environmental behavior. Following stewardship theory, employees consider themselves as stewards who are expected to take every initiative to preserve nature and pass on it to the next generations without destroying it. Therefore, we find this theory as an alternative for explaining employees’ engagement in pro-environmental behavior. More specifically, the concept of stewardship is well-transmitted in employees when they work for a socially responsible organization. This is because the very engagement of their organization in socially responsible activities inculcates in the employees that their organization is working as a steward and preserving nature for the future generation. They also are self-convinced that they should act as a steward and should work for the greater good of others.

### 5.1. Implications

The current analysis offers some important implications for theory and practice. In this regard, the first theoretical implication of the current study is that it is one of those few studies that stress to explain why employees are involved in pro-environmental behavior. Unlike the majority of the previous studies in this domain, which were carried out to explore the outputs of pro-environmental behavior [8,21], this study uncovers the underlying psychological mechanism of employees’ engagement in pro-environmental behavior. Another theoretical implication of the current study is that it is one of the pioneering studies that attempt to link the concept of work engagement to explain PEB-E. The phenomenon of work engagement as a mediator has received significant attention from various scholars to explain employees’ extra-role behavior, for example, employee creativity [103,106] and employee innovation capability [107,108]. Employees’ pro-environmental behavior is also an extra-role, but surprisingly, contemporary researchers did not explore the effect of work engagement on pro-environmental behavior. We found only one recent study in the available literature from Raza et al. [20], who validated the mediating effect of work engagement to explain employees’ pro-environmental behavior. Yet, another theoretical implication of the current study is that it employs stewardship theory as an alternate theory for explaining employees’ engagement in pro-environmental behavior. Whereas, other scholars have largely linked social identity theory, social learning theory, and social exchange theory to explain employees’ pro-environmental behavior. 

The practical implications of the current study are equally important to mention here. First of all, the current study attempts to highlight the importance of CSR from the perspective of employees’ extra-role behavior. In this regard, it is important to mention here that the majority of the available literature has linked CSR to achieving different organizational objectives, including performance, reputation, and organizational image. The current study uncovers the importance of CSR for the policymakers of the SME sector of emerging economy from a standpoint of employees. The study argues that through better employees’ CSR perception, an SME can engage its employees into different extra roles, one of which is pro-environmental behavior. This finding has a special meaning for the SME sector which is largely characterized as a sector with scarce financial resources. SMEs have been consistently reported to be resource-deficient entities. In this regard, the findings of the current study are of utmost importance for the SME sector in a sense that employees’ pro-environmental behavior means that employees will not be wasting organizational resources, rather they will be taking care of these resources. Employees who use an SME’s resources efficiently implies that such SME will be in a better position from the perspective of resource availability. On this basis, SMEs can have a better hope of survivability if they are socially responsible. 

Another important practical implication of the current study is that it highlights the importance of work engagement to achieve the sustainability objectives of an organization. Unfortunately, the SME sector of is less oriented towards work engagement. Although the sector realizes the importance of work engagement, it is slightly unaware of the factors that can engage workers more readily. In this regard, the current study offers this sector a fresh insight to update their understanding of how to enhance the engagement of their workers. Last, the current study has another practical implication, as the findings of the current study are helpful in improving the understanding of the policymakers from the SME sector to think CSR beyond the philanthropic boundary as currently, the majority of SME assumes CSR to the extent of philanthropic responsibility. The policymakers need to realize that philanthropy is only one aspect of CSR whereas the whole philosophy of CSR is multitude and has many benefits for an enterprise.

### 5.2. Limitations and Future Directions

The current study is not without limitations. Although, these limitations open new horizons for upcoming researchers in the same field. The first limitation of the current study is that it collected the data from only one city due to the restricted movement in the age of the COVID-19 pandemic. However, we think that the geographical boundary should be extended in future studies in order to have better generalizability. Another important observation is that this study attempts to explain employees’ behavior, especially their extra-role behavior through CSR and W.E. Although, the proposed relationships were significant, we still consider that human behavior is very complex to understand and explaining such complexity with the help of two variables may have specific consequences. In this regard, we suggest future researchers add more variables to the research model of the current study. The cross-sectional data design of the current survey is also a limitation as cross-sectional data meets its boundary condition in explaining the causal relationships. Therefore, for future studies, we propose a longitudinal dataset to be included, if possible. Finally, in the present study, the influence of demographic variables on the proposed framework was not explored. However, it is indubitable that such demographic variables are important in the formation of employee pro-environmental behaviors. Therefore, testing the effect of demographic factors will be a useful direction for future studies. 

## 6. Conclusions

Despite the positive contribution of SMEs, their activities are a major cause of environmental problems. The pro-environmental behavior of employees is an under-researched area in the context of SMEs. To this end, the current study investigated the relationship between CSR and pro-environmental behavior of employees with the mediating effect of W.E in the SME sector. The empirical findings of the current survey unveiled that the CSR efforts of an SME are well-observed by its employees, and they are self-enhanced to support their SME in achieving its sustainability objectives. Further, as the CSR commitment is assumed as an extra-commitment of an organization, the employees also copy this commitment and put forth their every effort to preserve the natural environment for future generations. They consequently change their habitual behavior at the workplace to pro-environmental behavior. The role of W.E also helps to explain the above relationship in a way that the engaged employees bring their whole selves to the workplace, and they are well committed to support their organization. Furthermore, the management of SMEs is suggested to endeavor to bring nature into the workplace as humans (including employees) have an intrinsic need to be connected with nature. For example, SMEs need to arrange training and seminars with a special focus to promote sustainable behavior among employees. In this regard, it will also be important if SMEs convert their outlay to an environment-friendly organization along with its CSR activities. This can be done by incorporating nature into the workplace, e.g., offices with green walls, green buildings, indoor trees and planter boxes, etc. On a final note, as the SME sector is a labor-intensive sector in a country, and if the employees in this sector can show a better commitment to preserve nature, the country may hope to achieve a better and sustainable future. 

## Figures and Tables

**Table 1:** Sample profile.

Age	Frequency (*n* = 398)	Gender	Frequency (*n* = 398)
18–25	74	Male	241
26–30	89	Female	157
31–35	107		
36–40	82		
Above 40	46		
Experience		Industry	
		Plastic products	75
1–3	79	Textiles	93
4–6	117	Cosmetics	48
7–10	113	Auto Parts	59
Above	89	Chemicals	62
		Food Processing	61

**Table 2:** Output of single-factor analysis.

Total Variance Explained						
Factor	Initial Eigenvalues			Extraction Sums of Squared Loadings		
	Total	% of Variance	Cumulative %	Total	% of Variance	Cumulative %
1	7.125	42.097	42.097	6.381	40.165	40.165
2	2.596	9.615	36.005			
3	2.039	7.551	43.557			
4	1.456	5.394	48.951			
5	0.984	4.657	53.608			
6	0.988	3.874	57.482			
7	0.962	3.564	61.046			
8	0.837	3.100	64.146			
9	0.786	2.913	67.058			
10	0.761	2.818	69.877			
11	0.726	2.688	72.565			
12	0.698	2.585	75.149			
13	0.654	2.422	77.572			
14	0.623	2.308	79.880			
15	0.587	2.175	82.054			
16	0.562	2.082	84.136			
17	0.545	2.019	86.155			
18	0.508	1.883	88.038			
19	0.488	1.806	89.844			
20	0.443	1.640	91.483			
21	0.441	1.632	93.115			
22	0.375	1.389	94.505			
23	0.368	1.363	95.868			
24	0.339	1.257	97.124			
25	0.325	1.202	98.327			
26	0.280	1.036	99.363			
27	0.172	0.637	100.000			

**Note:** Extraction Method: Principal Axis Factoring without any rotation.

**Table 3:** Item loadings, convergent validity and reliability results.

Item	λ	λ^2^	E-Variance	∑λ^2^	Items	AVE	C.R
CSR-1	0.78	0.61	0.39				
CSR-2	0.82	0.67	0.33				
CSR-3	0.70	0.49	0.51				
CSR-4	0.68	0.46	0.54				
CSR-5	0.74	0.55	0.45				
CSR-6	0.86	0.74	0.26				
CSR-7	0.70	0.49	0.51				
CSR-8	0.73	0.53	0.47				
CSR-9	0.71	0.50	0.50				
CSR-10	0.87	0.76	0.24				
CSR-11	0.81	0.66	0.34				
CSR-12	0.72	0.52	0.48	6.98	12	0.76	0.94
W.E-1	0.82	0.67	0.33				
W.E-2	0.76	0.58	0.42				
W.E-3	0.72	0.52	0.48				
W.E-4	0.83	0.69	0.31				
W.E-5	0.80	0.64	0.36				
W.E-6	0.77	0.59	0.41				
W.E-7	0.74	0.55	0.45				
W.E-8	0.71	0.50	0.50				
W.E-9	0.78	0.61	0.39	5.35	9	0.59	0.93
PEB-E-1	0.73	0.53	0.47				
PEB-E-2	0.76	0.58	0.42				
PEB-E-3	0.71	0.50	0.50				
PEB-E-4	0.79	0.62	0.38				
PEB-E-5	0.88	0.77	0.22				
PEB-E-6	0.82	0.67	0.33	3.69	6	0.61	0.90

**Notes:** λ = Item loadings, C.R = composite reliability, ∑λ^2^ = sum of square of item loadings, E-Variance = error variance.

**Table 4:** Correlation, discriminant validity and model fit indices.

Construct	CSR	W.E	PEB-E
CSR	**0.88**	0.39 **	0.41 **
W.E		**0.93**	0.33 **
PEB-E			**0.90**
Mean	3.78	4.11	4.23
SD	0.70	0.52	0.53
MSV	0.17	0.15	0.17
ASV	0.16	0.14	0.14
HTMT	0.82	0.79	0.84

**Notes:** S.D = standard deviation, ** = significant values of correlation, bold diagonal = Cronbach alpha, maximum shared variance = MSV, and average shared variance = ASV.

**Table 5:** The results for hypotheses testing (H1, H2 and H3).

Path	Estimates	S.E	CR	*p*-Value	ULCI	LLCI	Decision
CSR → PEB-E	(*β*1) 0.36 **	0.046	7.83	***	0.427	0.280	Approved
CSR → W.E	(*β2*) 0.32 **	0.052	6.15	***	0.397	0.311	Approved
W.E → PEB-E	(*β2*) 0.26 **	0.038	6.84	***	0.388	0.293	Approved

**Notes:** ULCI = upper-limit confidence interval, LLCI = lower-limit confidence interval, **, *** = significant values.

**Table 6:** Mediation results for H4.

Path	Estimates	S.E	Z-Score	*p*-Value	ULCI	LLCI	*R^2^*	Decision
CSR → W.E→ PEB-E	(*β4*) 0.083 **	0.012	6.93	***	0.188	0.116	0.26	Approved

**Notes**: ULCI = upper-limit confidence interval, LLCI = lower-limit confidence interval, **, *** = significant values, S.E = standard error.

## Data Availability

The data will be made available on request.

## Appendix A

**Table A1:** Mean and standard deviation of items.

Item	Mean	Std.	Code
1-Our company participates in activities, which aim to protect and improve the quality of the natural environment	3.92	0.837	CSR1
1-I make suggestions about environmentally friendly practices	4.13	0.804	PEB1
4-Our company makes investment to create a better life for future generations	3.70	0.682	CSR4
5-I am immersed in my work	4.04	0.527	WE5
9-I get carried away when I’m working	3.95	0.485	WE9
2-Our company implements special programs to minimize its negative impact on the natural environment	3.40	0.687	CSR2
6-I take part in environmentally friendly programs (e.g., bike/walk to work day, bring own local lunch daily)	4.32	0.521	PEB6
7-I am proud on the work that I do	4.25	0.820	WE7
3-Our company targets sustainable growth, which considers future generations	3.43	0.555	CSR3
8-Our company supports nongovernmental organizations working in problematic areas	3.78	0.498	CSR8
3-I turn lights off when not in use	4.05	0.846	PEB3
10-Our company contributes to campaigns and projects that aim to promote the well-being of the society	4.05	0.782	CSR10
4-I feel happy when I am working intensely	4.03	0.630	WE4
12-Our company encourages its employees to participate in voluntary activities	3.78	0.833	CSR12
9-Our company respects consumer rights beyond the legal requirements	4.18	0.757	CSR9
6-When I get up in the morning, I feel like going to work	4.91	0.284	WE6
1-My job inspires me	3.58	0.644	WE1
2-I bring reusable eating utensils to work (e.g., travel coffee mug, water bottle, reusable containers, reusable cutlery)	4.18	0.490	PEB2
7-Our company provides full and accurate information about its product to its customers	3.73	0.452	CSR7
8-Customer satisfaction is highly important for our company	4.17	0.811	CSR6
3-I am enthusiastic about my job	3.98	0.642	WE3
2-At my job, I feel strong and vigorous	4.06	0.389	WE2
5-I put compostable items in the compost bin PEB5	4.19	0.653	PEB5
5-Our company always pays its taxes on a regular and continuing basis	3.47	0.712	CSR5
4-I print double sided whenever possible	4.54	0.583	PEB4
11-Our company complies with legal regulations completely and promptly	3.80	0.884	CSR11
8-At my work, I feel bursting with energy	4.14	0.318	WE8

## References

[1] Journeault M., Perron A., Vallières L. (2021). The collaborative roles of stakeholders in supporting the adoption of sustainability in SMEs. J. Environ. Manag..

[2] Rodrigues da Costa L., Maria Correia Loureiro S. (2019). The importance of employees’ engagement on the organizational success. J. Promot. Manag..

[3] Kim Y.J., Kim W.G., Choi H.-M., Phetvaroon K. (2019). The effect of green human resource management on hotel employees’ eco-friendly behavior and environmental performance. Int. J. Hosp. Manag..

[4] Gilal F.G., Ashraf Z., Gilal N.G., Gilal R.G., Channa N.A. (2019). Promoting environmental performance through green human resource management practices in higher education institutions: A moderated mediation model. Corp. Soc. Responsib. Environ. Manag..

[5] Anwar N., Mahmood N.H.N., Yusliza M.Y., Ramayah T., Faezah J.N., Khalid W. (2020). Green Human Resource Management for organisational citizenship behaviour towards the environment and environmental performance on a university campus. J. Clean. Prod..

[6] Murtaza S.A., Mahmood A., Saleem S., Ahmad N., Sharif M.S., Molnár E. (2021). Proposing Stewardship Theory as an Alternate to Explain the Relationship between CSR and Employees’ Pro-Environmental Behavior. Sustainability.

[7] Afsar B., Umrani W.A. (2020). Corporate social responsibility and pro-environmental behavior at workplace: The role of moral reflectiveness, coworker advocacy, and environmental commitment. Corp. Soc. Responsib. Environ. Manag..

[8] Ahmad N., Ullah Z., Arshad M.Z., waqas Kamran H., Scholz M., Han H. (2021). Relationship between corporate social responsibility at the micro-level and environmental performance: The mediating role of employee pro-environmental behavior and the moderating role of gender. Sustain. Prod. Consum..

[9] Shah S.H.A., Cheema S., Al-Ghazali B.M., Ali M., Rafiq N. (2021). Perceived corporate social responsibility and pro-environmental behaviors: The role of organizational identification and coworker pro-environmental advocacy. Corp. Soc. Responsib. Environ. Manag..

[10] Ahmad N., Mahmood A., Han H., Ariza-Montes A., Vega-Muñoz A., Iqbal Khan G., Ullah Z. (2021). Sustainability as a “new normal” for modern businesses: Are smes of pakistan ready to adopt it?. Sustainability.

[11] Moon J. (2007). The contribution of corporate social responsibility to sustainable development. Sustain. Dev..

[12] Marano V., Kostova T. (2016). Unpacking the institutional complexity in adoption of CSR practices in multinational enterprises. J. Manag. Stud..

[13] Carroll A.B. (1991). The pyramid of corporate social responsibility: Toward the moral management of organizational stakeholders. Bus. Horiz..

[14] Pinkston T.S., Carroll A.B. (1996). A retrospective examination of CSR orientations: Have they changed?. J. Bus. Ethics.

[15] Fauzi H., Idris K. (2009). The relationship of CSR and financial performance: New evidence from Indonesian companies. Issues Soc. Environ. Account..

[16] Torugsa N.A., O’Donohue W., Hecker R. (2012). Capabilities, proactive CSR and financial performance in SMEs: Empirical evidence from an Australian manufacturing industry sector. J. Bus. Ethics.

[17] Inoue Y., Kent A., Lee S. (2011). CSR and the bottom line: Analyzing the link between CSR and financial performance for professional teams. J. Sport Manag..

[18] Kim H.-S. (2011). A reputational approach examining publics’ attributions on corporate social responsibility motives. Asian J. Commun..

[19] Sotorrío L.L., Sánchez J.L.F. (2008). Corporate social responsibility of the most highly reputed European and North American firms. J. Bus. Ethics.

[20] Raza A., Farrukh M., Iqbal M.K., Farhan M., Wu Y. (2021). Corporate social responsibility and employees’ voluntary pro-environmental behavior: The role of organizational pride and employee engagement. Corporate Social Responsibility and Environmental Management.

[21] Nisar Q.A., Haider S., Ali F., Jamshed S., Ryu K., Gill S.S. (2021). Green Human Resource Management Practices and Environmental Performance in Malaysian Green Hotels: The role of Green Intellectual Capital and Pro-Environmental Behavior. J. Clean. Prod..

[22] Elshaer I.A., Sobaih A.E.E., Aliedan M., Azzaz A. (2021). The Effect of Green Human Resource Management on Environmental Performance in Small Tourism Enterprises: Mediating Role of Pro-Environmental Behaviors. Sustainability.

[23] GermanWatch Global Climate Risk Index 2020. https://www.germanwatch.org/.

[24] Ajmal M.M. Industrial Pollution in Pakistan. https://nation.com.pk/07-Jan-2019/industrial-pollution-in-pakistan.

[25] Zou Z., Liu Y., Ahmad N., Sial M.S., Badulescu A., Zia-Ud-Din M., Badulescu D. (2021). What Prompts Small and Medium Enterprises to Implement CSR? A Qualitative Insight from an Emerging Economy. Sustainability.

[26] Tang Z., Tang J. (2012). Stakeholder–firm power difference, stakeholders’ CSR orientation, and SMEs’ environmental performance in China. J. Bus. Ventur..

[27] Barauskaite G., Streimikiene D. (2020). Corporate social responsibility and financial performance of companies: The puzzle of concepts, definitions and assessment methods. Corp. Soc. Responsib. Environ. Manag..

[28] Belu C., Manescu C. (2013). Strategic corporate social responsibility and economic performance. Appl. Econ..

[29] Halme M., Rintamäki J., Knudsen J.S., Lankoski L., Kuisma M. (2020). When is there a sustainability case for CSR? Pathways to environmental and social performance improvements. Bus. Soc..

[30] Abbas J. (2020). Impact of total quality management on corporate green performance through the mediating role of corporate social responsibility. J. Clean. Prod..

[31] Chaudhary R., Akhouri A. (2019). CSR perceptions and employee creativity: Examining serial mediation effects of meaningfulness and work engagement. Soc. Responsib. J..

[32] Sulea C., Virga D., Maricutoiu L.P., Schaufeli W., Zaborila Dumitru C., Sava F.A. (2012). Work engagement as mediator between job characteristics and positive and negative extra-role behaviors. Career Dev. Int..

[33] Donaldson L., Davis J.H. (1991). Stewardship theory or agency theory: CEO governance and shareholder returns. Aust. J. Manag..

[34] Pastoriza D., Ariño M.A. When agents become stewards: Introducing learning in the stewardship theory. Proceedings of the 1st IESE Conference, “Humanizing the Firm & Management Profession”.

[35] Eddleston K.A., Kellermanns F.W. (2007). Destructive and productive family relationships: A stewardship theory perspective. J. Bus. Ventur..

[36] Subramanian S. (2018). Stewardship theory of corporate governance and value system: The case of a family-owned business group in India. Indian J. Corp. Gov..

[37] Madhani P.M. (2017). Diverse roles of corporate board: Review of various corporate governance theories. IUP J. Corp. Gov..

[38] Larbi M. (2014). Environmental Stewardship and Corporate Social Responsibility: Implication for Consumers’ Resistance to Negative Information. Case Apple China.

[39] Kuttner M., Feldbauer-Durstmüller B., Mitter C. (2021). Corporate social responsibility in Austrian family firms: Socioemotional wealth and stewardship insights from a qualitative approach. J. Fam. Bus. Manag..

[40] Bormann K.C., Backs S., Hoon C. (2020). What Makes Nonfamily Employees Act as Good Stewards? Emotions and the Moderating Roles of Stewardship Culture and Gender Roles in Family Firms. Fam. Bus. Rev..

[41] McWilliams A., Siegel D. (2001). Corporate social responsibility: A theory of the firm perspective. Acad. Manag. Rev..

[42] Lu J., Ren L., Zhang C., Wang C., Ahmed R.R., Streimikis J. (2020). Corporate social responsibility and employee behavior: Evidence from mediation and moderation analysis. Corp. Soc. Responsib. Environ. Manag..

[43] Ahmed M., Zehou S., Raza S.A., Qureshi M.A., Yousufi S.Q. (2020). Impact of CSR and environmental triggers on employee green behavior: The mediating effect of employee well-being. Corp. Soc. Responsib. Environ. Manag..

[44] Shen J., Benson J. (2016). When CSR is a social norm: How socially responsible human resource management affects employee work behavior. J. Manag..

[45] Wisse B., van Eijbergen R., Rietzschel E.F., Scheibe S. (2018). Catering to the needs of an aging workforce: The role of employee age in the relationship between corporate social responsibility and employee satisfaction. J. Bus. Ethics.

[46] Yu H., Shabbir M.S., Ahmad N., Ariza-Montes A., Vega-Muñoz A., Han H., Scholz M., Sial M.S. (2021). A contemporary issue of micro-foundation of CSR, employee pro-environmental behavior, and environmental performance toward energy saving, carbon emission reduction, and recycling. Int. J. Environ. Res. Public Health.

[47] Kollmuss A., Agyeman J. (2002). Mind the gap: Why do people act environmentally and what are the barriers to pro-environmental behavior?. Environ. Educ. Res..

[48] Afsar B., Badir Y., Kiani U.S. (2016). Linking spiritual leadership and employee pro-environmental behavior: The influence of workplace spirituality, intrinsic motivation, and environmental passion. J. Environ. Psychol..

[49] Sial M., Zheng C., Cherian J., Gulzar M., Thu P., Khan T., Khuong N. (2018). oes Corporate Social Responsibility Mediate the Relation between Boardroom Gender Diversity and Firm Performance of Chinese Listed Companies?. Sustainability.

[50] Hameed Z., Khan I.U., Islam T., Sheikh Z., Khan S.U. (2019). Corporate social responsibility and employee pro-environmental behaviors. South Asian J. Bus. Stud..

[51] Tian Q., Robertson J.L. (2019). How and when does perceived CSR affect employees’ engagement in voluntary pro-environmental behavior?. J. Bus. Ethics.

[52] Suganthi L. (2019). Examining the relationship between corporate social responsibility, performance, employees’ pro-environmental behavior at work with green practices as mediator. J. Clean. Prod..

[53] Islam T., Ali G., Asad H. (2019). Environmental CSR and pro-environmental behaviors to reduce environmental dilapidation. Manag. Res. Rev..

[54] Alzaidi S.M., Iyanna S. (2021). Developing a conceptual model for voluntary pro-environmental behavior of employees. Soc. Responsib. J..

[55] Kaliannan M., Adjovu S.N. (2015). Effective employee engagement and organizational success: A case study. Procedia-Soc. Behav. Sci..

[56] Cesário F., Chambel M.J. (2017). Linking organizational commitment and work engagement to employee performance. Knowl. Process Manag..

[57] GALLUP 2018 Global Great Jobs Briefing. https://news.gallup.com/reports/233375/gallup-global-great-jobs-report-2018.aspx.

[58] Kim W., Park J. (2017). Examining structural relationships between work engagement, organizational procedural justice, knowledge sharing, and innovative work behavior for sustainable organizations. Sustainability.

[59] Kodden B. (2020). The Relationship Between Work Engagement and Sustainable Performance. The Art of Sustainable Performance.

[60] Wellins R.S., Bernthal P., Phelps M. (2005). Employee engagement: The key to realizing competitive advantage. Dev. Dimens. Int..

[61] Nazir O., Islam J.U. (2020). Effect of CSR activities on meaningfulness, compassion, and employee engagement: A sense-making theoretical approach. Int. J. Hosp. Manag..

[62] Hur W.-M., Moon T.-W., Ko S.-H. (2018). How employees’ perceptions of CSR increase employee creativity: Mediating mechanisms of compassion at work and intrinsic motivation. J. Bus. Ethics.

[63] Glavas A. (2016). Corporate social responsibility and employee engagement: Enabling employees to employ more of their whole selves at work. Front. Psychol..

[64] Chaudhary R., Akhouri A. (2018). CSR Attributions, Work engagement and Creativity: Examining the role of Authentic Leadership. AOM J..

[65] Fatima J.K., Di Mascio R., Sharma P. (2020). Demystifying the impact of self-indulgence and self-control on customer-employee rapport and customer happiness. J. Retail. Consum. Serv..

[66] Tworzydło D., Gawroński S., Szuba P. (2021). Importance and role of CSR and stakeholder engagement strategy in polish companies in the context of activities of experts handling public relations. Corp. Soc. Responsib. Environ. Manag..

[67] Perrin T. Closing the Engagement Gap: A Road Map for Driving Superior Business Performance. https://engageforsuccess.org/wp-content/uploads/2015/10/Closing-the-engagement-gap-TowersPerrin.pdf.

[68] Parul B. Can CSR boost Employee Engagement?. https://www.peoplematters.in/article/talent-management/9-trends-that-will-shape-future-workplaces-16459.

[69] Albdour A.A., Altarawneh I.I. (2012). Corporate social responsibility and employee engagement in Jordan. Int. J. Bus. Manag..

[70] Glavas A., Piderit S.K. (2009). How does doing good matter? Effects of corporate citizenship on employees. J. Corp. Citizsh..

[71] Caligiuri P., Mencin A., Jiang K. (2013). Win–win–win: The influence of company-sponsored volunteerism programs on employees, NGOs, and business units. Pers. Psychol..

[72] Barrett S.M., Murphy D.F. (2017). Managing Corporate Environmental Policy: A Process of Complex Change. Greening People.

[73] Welford R. (2016). Corporate Environmental Management 1: Systems and Strategies.

[74] Joo B.-K., Lee I. (2017). Workplace happiness: Work engagement, career satisfaction, and subjective well-being. Evid.-Based HRM A Glob. Forum Empir. Sch..

[75] Kahn W.A. (1990). Psychological conditions of personal engagement and disengagement at work. Acad. Manag. J..

[76] Chung C.Y., Jung S., Young J. (2018). Do CSR activities increase firm value? Evidence from the Korean market. Sustainability.

[77] Sial M.S., Zheng C., Khuong N.V., Khan T., Usman M. (2018). Does Firm Performance Influence Corporate Social Responsibility Reporting of Chinese Listed Companies?. Sustainability.

[78] Farid T., Iqbal S., Ma J., Castro-González S., Khattak A., Khan M.K. (2019). Employees’ perceptions of CSR, work engagement, and organizational citizenship behavior: The mediating effects of organizational justice. Int. J. Environ. Res. Public Health.

[79] Brewer M.B. (2011). Optimal distinctiveness theory: Its history and development. Handb. Theor. Soc. Psychol..

[80] Steffens N.K., Haslam S.A., Reicher S.D., Platow M.J., Fransen K., Yang J., Ryan M.K., Jetten J., Peters K., Boen F. (2014). Leadership as social identity management: Introducing the Identity Leadership Inventory (ILI) to assess and validate a four-dimensional model. Leadersh. Q..

[81] Ahmad N., Ullah Z., Mahmood A., Ariza-Montes A., Vega-Muñoz A., Han H., Scholz M. (2021). Corporate social responsibility at the micro-level as a “new organizational value” for sustainability: Are females more aligned towards it?. Int. J. Environ. Res. Public Health.

[82] Popescu A.I., Aleksandra G. (2019). Learning by Engaging in Pro-Environmental Behaviour at Work. Pro-Ecological Restructuring of Companies|Case Studies.

[83] Tuan L.T. (2018). Driving employees to serve customers beyond their roles in the Vietnamese hospitality industry: The roles of paternalistic leadership and discretionary HR practices. Tour. Manag..

[84] Mai W. (2021). Short-Selling and Financial Performance of SMEs in China: The Mediating Role of CSR Performance. Int. J. Financ. Stud..

[85] Du J., Banwo A. (2015). Promoting sme competitiveness: Lessons from China and Nigeria. Am. Adv. Res. Manag..

[86] IQAir Air Quality in Pakistan. https://www.iqair.com/us/pakistan.

[87] Turker D. (2009). Measuring corporate social responsibility: A scale development study. J. Bus. Ethics.

[88] Schaufeli W.B., Bakker A.B., Salanova M. (2006). The measurement of work engagement with a short questionnaire: A cross-national study. Educ. Psychol. Meas..

[89] Robertson J.L., Barling J. (2013). Greening organizations through leaders’ influence on employees’ pro-environmental behaviors. J. Organ. Behav..

[90] Latif M.A., Aziz M.S. (2018). Workplace spirituality and pro-environmental behavior: The role of employee engagement and environmental awareness. Glob. J. Manag. Bus. Res..

[91] Afsar B., Cheema S., Javed F. (2018). Activating employee’s pro-environmental behaviors: The role of CSR, organizational identification, and environmentally specific servant leadership. Corp. Soc. Responsib. Environ. Manag..

[92] Podsakoff P.M., Organ D.W. (1986). Self-reports in organizational research: Problems and prospects. J. Manag..

[93] Harman H.H. (1976). Modern Factor Analysis.

[94] Fornell C., Larcker D.F. (1981). Evaluating structural equation models with uno bservable variables and measurement error. J. Mark. Res..

[95] Gefen D., Straub D., Boudreau M.-C. (2000). Structural equation modeling and regression: Guidelines for research practice. Commun. Assoc. Inf. Syst..

[96] Hair J., Anderson R., Babin B., Black W. (2010). Multivariate Data Analysis: A Global Perspective.

[97] Henseler J., Ringle C.M., Sarstedt M. (2015). A new criterion for assessing discriminant validity in variance-based structural equation modeling. J. Acad. Mark. Sci..

[98] Wheaton B., Muthen B., Alwin D.F., Summers G.F. (1977). Assessing reliability and stability in panel models. Sociol. Methodol..

[99] Richter N.F., Schubring S., Hauff S., Ringle C.M., Sarstedt M. (2020). When predictors of outcomes are necessary: Guidelines for the combined use of PLS-SEM and NCA. Ind. Manag. Data Syst..

[100] Matthews L. (2017). Applying multigroup analysis in PLS-SEM: A step-by-step process. Partial Least Squares Path Modeling.

[101] Thakkar J.J. (2020). Applications of structural equation modelling with AMOS 21, IBM SPSS. Structural Equation Modelling.

[102] Abdelmotaleb M., Mohamed Metwally A.B.E., Saha S.K. (2018). Exploring the impact of being perceived as a socially responsible organization on employee creativity. Manag. Decis..

[103] Asif M., Qing M., Hwang J., Shi H. (2019). Ethical leadership, affective commitment, work engagement, and creativity: Testing a multiple mediation approach. Sustainability.

[104] Gao Y., Zhang D., Huo Y. (2018). Corporate social responsibility and work engagement: Testing a moderated mediation model. J. Bus. Psychol..

[105] Tong Z., Zhu L., Zhang N., Livuza L., Zhou N. (2019). Employees’ perceptions of corporate social responsibility and creativity: Employee engagement as a mediator. Soc. Behav. Personal. Int. J..

[106] Chaudhary R., Akhouri A. (2018). Linking corporate social responsibility attributions and creativity: Modeling work engagement as a mediator. J. Clean. Prod..

[107] Tian G., Zhang Z. (2020). Linking empowering leadership to employee innovation: The mediating role of work engagement. Soc. Behav. Personal. Int. J..

[108] Ge Y., Sun X. (2020). The relationship of employees’ strengths use and innovation: Work engagement as a mediator. Soc. Behav. Personal. Int. J..

